# Bio-oil based biorefinery strategy for the production of succinic acid

**DOI:** 10.1186/1754-6834-6-74

**Published:** 2013-05-08

**Authors:** Caixia Wang, Anders Thygesen, Yilan Liu, Qiang Li, Maohua Yang, Dan Dang, Ze Wang, Yinhua Wan, Weigang Lin, Jianmin Xing

**Affiliations:** 1 National Key Laboratory of Biochemical Engineering, Institute of Process Engineering, Chinese Academy of Sciences, P. O. Box 353, No. 1 Zhongguancun North Second Street, Beijing 100190, P.R. China; 2 2University of Chinese Academy of Sciences, Beijing 100049, R.P. China; 3 Department of Chemical and Biochemical Engineering, Technical University of Denmark, Lyngby, DK-2800, Denmark; 4 Sino-Danish Center for Education and Research, Niels Jensensvej 2, DK-8000, Aarhus C, Denmark; 5 State Key Laboratory of Multiple Complex Systems, Institute of Process Engineering, Chinese Academy of Sciences, No. 1 Zhongguancun North Second Street, P. O. Box 353, , Beijing 100190, P.R. China

## Abstract

**Background:**

Succinic acid is one of the key platform chemicals which can be produced via biotechnology process instead of petrochemical process. Biomass derived bio-oil have been investigated intensively as an alternative of diesel and gasoline fuels. Bio-oil could be fractionized into organic phase and aqueous phase parts. The organic phase bio-oil can be easily upgraded to transport fuel. The aqueous phase bio-oil (AP-bio-oil) is of low value. There is no report for its usage or upgrading via biological methods. In this paper, the use of AP-bio-oil for the production of succinic acid was investigated.

**Results:**

The transgenic *E. coli* strain could grow in modified M9 medium containing 20 v/v% AP-bio-oil with an increase in OD from 0.25 to 1.09. And 0.38 g/L succinic acid was produced. With the presence of 4 g/L glucose in the medium, succinic acid concentration increased from 1.4 to 2.4 g/L by addition of 20 v/v% AP-bio-oil. When enzymatic hydrolysate of corn stover was used as carbon source, 10.3 g/L succinic acid was produced. The obtained succinic acid concentration increased to 11.5 g/L when 12.5 v/v% AP-bio-oil was added. However, it decreased to 8 g/L when 50 v/v% AP-bio-oil was added. GC-MS analysis revealed that some low molecular carbon compounds in the AP-bio-oil were utilized by *E. coli*.

**Conclusions:**

The results indicate that AP-bio-oil can be used by *E. coli* for cell growth and succinic acid production.

## Background

Because of increasing concerns of the exhausting resource and ecological and environmental problems in the petro-based industry, utilization of renewable resources is considered as one of the solutions for sustainable development. Biomass is one of the most abundant and most important renewable resources, which can be used as feedstocks to produce energy, platform chemicals and materials in biorefinery [1]. Biorefinery is a promising concept as an alternative to petro-based refinery industry.

Succinic acid, a four-carbon dicarboxylic acid produced as an intermediate of the tricarboxylic acid cycle or as an end product of anaerobic metabolism, has been widely used in the agricultural, food and pharmaceutical industries [2]. Currently, succinic acid is considered as one of the key platform chemicals used directly in preparation of biodegradable polymers such as polybutylene succinate and polyamides and as a raw material to synthesize compounds in the C4 family, including 1,4-butanediol, tetrahydrofuran, N-methyl pyrolidinone, 2-pyrrolidinone and γ-butyrolactone [3,4]. Due to its independence from petroleum as a raw material, environmental benefit and CO_2_ sequestration, biological production of succinic acid from renewable resources has attracted significant interest over the recent years [5,6]. A wide variety of strains have been applied for the production of succinic acid, such as *Actinobacillus succinogenes *[7], *Mannheimia succiniciproducens *[8], *Anaerobiospirillum succiniciproducens *[9], and recombinant *Escherichia coli*[10]. Due to the well-understood physiology and the well-established engineering tools, *E. coli* has been studied intensively and has showed great advantages in succinic acid production such as a wide range of carbon sources and tolerance to the complicated environment [11]. So far, bio-succinic acid cannot compete with that derived from petro-based processes due to the high cost of the raw materials. In this situation, the biorefinery strategy opens a promising way for the production of succinic acid since cheaper biomass waste potentially can be utilized [12,13].

Biorefinery consist of two platforms: a sugar platform and a thermal platform. Nowadays, renewable biomass has been intensively investigated to produce bio-fuels and chemicals via the sugar platform [14]. This process usually includes pretreatment of biomass, obtaining sugars and the final products fermentation. Meanwhile, substantial research is being carried out to produce alternative fuels from biomass to replace the gasoline and diesel via thermal platform [15]. Fast pyrolysis is one of the promising thermal processes, which is conducted at a median temperature (400 – 600°C) in the absence of oxygen at a high heating rate [16,17]. Production of bio-oil by pyrolysis of biomass attracts large attention since it has a higher energy density and has potentials for partial replacement of diesel and gasoline fuels [18]. However, the bio-oil cannot be used directly as transportation fuel due to its high oxygen content (40–50 w/w%), the low H/C ratios and the high water content (15–30 w/w%). Upgrading technologies such as deep deoxygenation is essential to promote the usage of bio-oil [19]. Bio-oil can be separated into two fractions after adding water, a heavy organic fraction and an aqueous fraction (AP-bio-oil). The heating value of pyrolytic lignin which is derived from the organic fraction is higher than the crude bio-oil because of its lower oxygen content [20,21]. Thus, it seems to be a good way to separate bio-oil into an aqueous phase and an organic phase before upgrading it [22]. It is reported that the AP-bio-oil contains many different components with the “sugar constituents” being a major part [23,24]. However, the concentrations of the components are low and are difficult to upgrade to a useful fuel. The possibility of AP-bio-oil usage was therefore investigated, that is, by applying a biotechnological process. It is of significant value to transform AP- bio-oil into value added chemicals. From authors’ knowledge, production of succinic acid from bio-oil via biological processes is not found in the open literature.

In this study, bio-oil was separated into an organic phase which can be easily upgraded to transportation fuel and an aqueous phase with water soluble organic components. The influence of AP- bio-oil on the bacterial growth and fermentation was investigated. Meanwhile glucose from enzymatic hydrolyzed corn stover was used to facilitate better fermentation process. This study thereby integrates the two biorefinery platforms with focus on production of succinic acid from bio-oil, which provides new insight into production of succinic acid from AP-bio-oil and corn stover.

## Results and discussion

**Figure 1:**
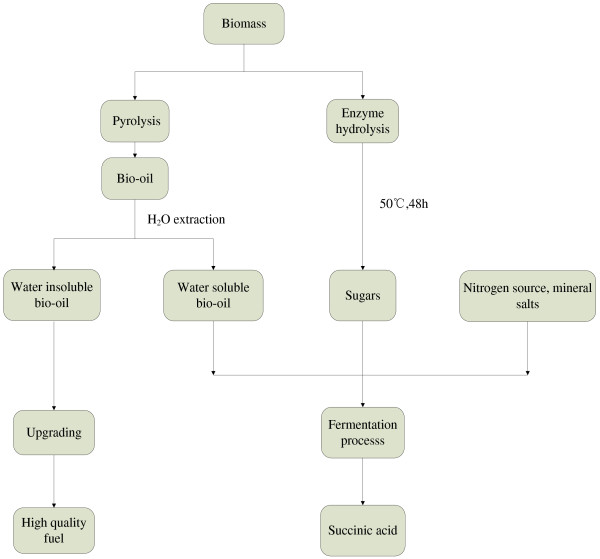
Co-production of high quality bio-oil and succinic acid from biomass treated by both thermochemical and biotechnological processes.

This study focused on the succinic acid production from bio-oil and enzyme hydrolysates as outlined in Figure 1. After pyrolysis, bio-oil was obtained and then phase fractionation was applied to obtain AP-bio-oil. AP-bio-oil and carbohydrates obtained from enzymatic hydrolyzed corn stover were used for succinic acid production using the *E.coli* strain MG-PYC constructed in this study. The influence of the AP-bio-oil on the bacterial growth and fermentation was thereby characterized.

### Bacterial growth

Bio-oil contains hundreds of compounds including carboxylic acids, alcohols, aldehydes, ketones, phenols, guaiacols, syringols, carbohydrates, furans, alkenes, aromatics, nitrogen containing organic compounds, and miscellaneous oxygenates [25]. Compounds dissolved in AP-bio-oil were mainly carbohydrate derived such as carboxylic acids and low-molecular-weight compounds. Acetic acid is identified as the most abundant compound in AP-bio-oil and the following one is formic acid [26].

**Figure 2:**
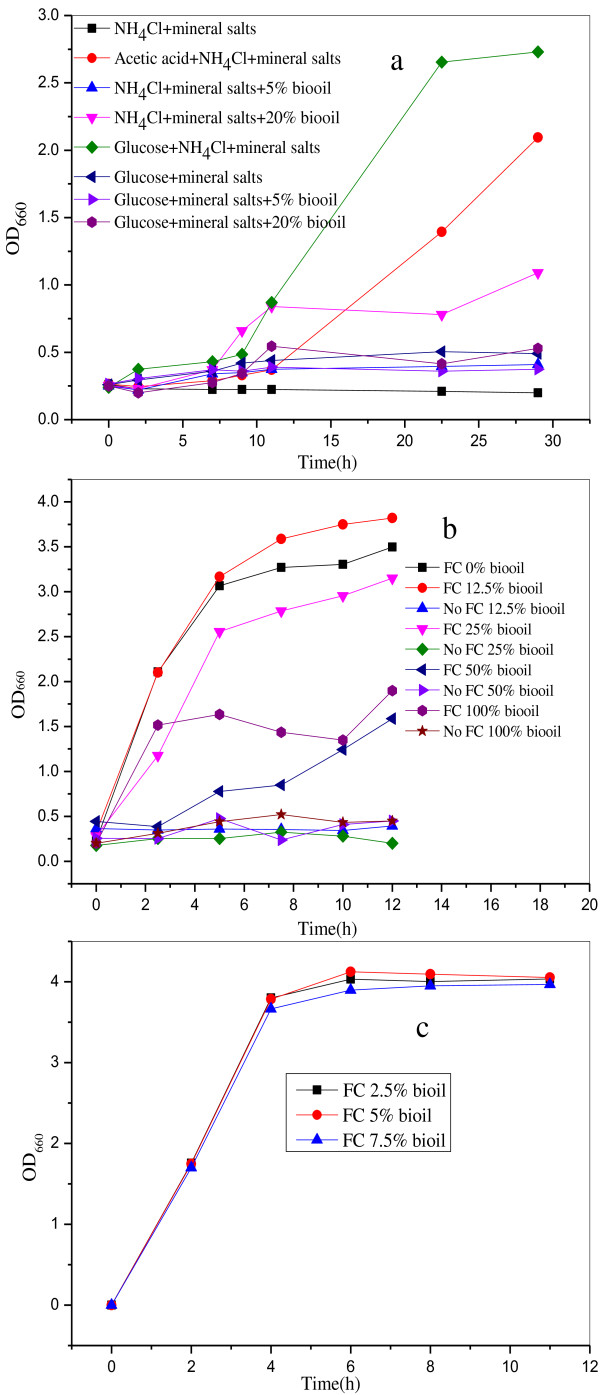
**Bacterial growth in test of the media. a**: bacterial growth in modified M9 media; **b**: bacterial growth in the traditional fermentation media with or without Fermentation components; **c**: bacterial growth in the traditional fermentation media with low concentrations of AP-bio-oil.

Figure 2 shows the growth of the *E. coli* MG-PYC strain with the tested media. Figure 2 (a) indicated the growth in modified M9 medium, according to the experimental design shown in Table 1. Besides glucose, acetic acid can be a carbon source for this strain because the OD value increased from 0.26 to 2.1 (Figure 2). For the medium which contains NH_4_Cl, mineral salts and 20 v/v% AP-bio-oil (medium 4), the OD value increased from 0.25 to 1.09. It illustrates that the bacteria grows better than in a similar medium with 5 v/v% AP-bio-oil. Without AP-bio-oil and glucose (medium 1), the OD did not increase which illustrates that there was no bacterial growth. From these results, it can be deduced that AP-bio-oil can provide carbon source to support the growth of this strain. By comparison of media 6, 7 and 8, the OD values increased very little which mean that the AP-bio-oil provides insufficient nitrogen source to support the bacterial growth. It has been reported that in AP-bio-oil, the carbon content is 52 w/w%, and the nitrogen content is 1 w/w% [27]. It is therefore expected that aqueous phase AP-bio-oil is a poor nitrogen source. Although AP-bio-oil can provide some carbon source, for a better fermentation process the bacterium needs additional nutrition such as protein and mineral salts. Figure 2 (b) shows the bacterial growth under the different percentages of AP-bio-oil with and without Fermentation components as stated in part 4.1. This graph shows that the strain grows better as the AP-bio-oil percentage decreased, which means that some inhibitory compounds existed in the AP-bio-oil. This inhibitory effect was evident at AP-bio-oil concentrations above 25 v/v% since the bacterium grew better in the 12.5 v/v% AP-bio-oil (OD = 3.8) than in the absence of AP-bio-oil (OD = 3.4). This is in agreement with Figure 2 (c) which shows the OD_600_ values at AP-bio-oil concentrations between 2.5 and 7.5 v/v%. Figure 1 (b) also showed that without Fermentation components this strain cannot grow, which means that AP-bio-oil itself cannot be a complete medium for this bacterium.

**Table 1:** Strain growth and succinic acid fermentation in modified M9 medium

**Number**	**Media component**	**Strain growth (OD600)**	**Succinic acid(g/L)**
1	M9 minral salts + NH_4_Cl	0.26	-
2	M9 minral salts + Acetic acid + NH_4_Cl	2.09	-
3	M9 minral salts + NH_4_Cl + 5% AP-bio-oil	0.41	0.29 ± 0.02
4	M9 minral salts + NH_4_Cl +20% AP-bio-oil	1.09	0.38 ± 0.03
5	M9 minral salts + Glucose + NH_4_Cl	2.37	1.87 ± 0.20
6	M9 minral salts + Glucose	0.49	1.40 ± 0.04
7	M9 minral salts + Glucose +5% AP-bio-oil	0.37	1.54 ± 0.04
8	M9 minral salts + Glucose +20% AP-bio-oil	0.53	2.42 ± 0.09

“‐”means no growth or no products detected.

### Fermentative production of succinic acid with AP-bio-oil and fermentation components

**Figure 3:**
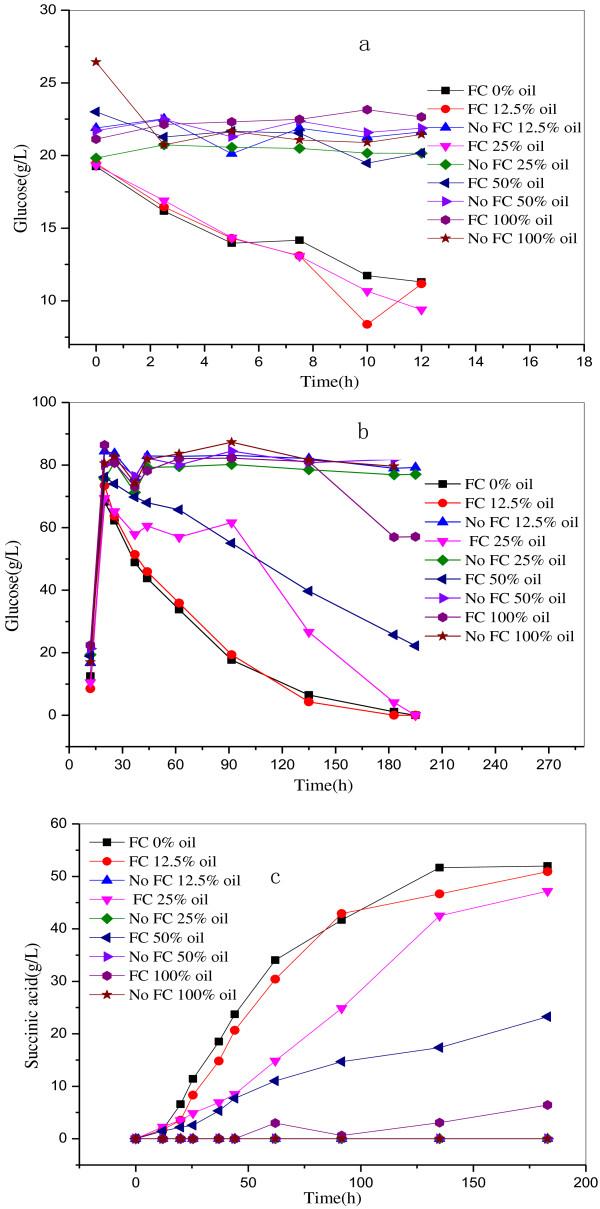
**Succinic acid production with different percentages of AP-bio-oil with or without Fermentation components. a**: glucose variation during the aerobic phase; **b**: glucose variation during the anaerobic phase; **c**: succinic acid fermentation during the anaerobic phase.

Pure AP-Bio-oil was tested to see if this can be fermented to succinic acid (Figure 3c). No succinic acid was produced in this experiment, since there are no mineral salts and insufficient nitrogen source in the AP-bio-oil. In the following work, modified M9 media was designed to test whether bio-oil can be utilized for the succinic acid fermentation as presented in Table 1. This resulted in succinic acid production which proved that AP-bio-oil with some mineral salts (NH_4_Cl) can provide the required nutrition. This usage of the AP-bio-oil for production of succinic acid is thereby meaningful.

However, succinic acid concentrations in Table 1 were not so similar. For the media without glucose (media 3 and 4), the final succinic acid concentrations of 0.29 g/L and 0.38 g/L are lower than in the media without NH_4_Cl but with glucose with 1.40 -2.42 g/L (media 6–8). It is notable that under these conditions, succinic acid concentration increased versus AP-bio-oil concentration in the range 0–20 v/v% with NH_4_Cl to 0.38 g/L and with glucose to 2.42 g/L. These results thereby indicate that AP-bio-oil can be used for succinic acid production. However, it is far from industrialization because of the low concentrations obtained. Thus, besides AP-bio-oil extra glucose must be added to obtain a sufficient succinic acid concentration.

In addition, different percentages of AP-bio-oil with and without Fermentation components were applied to test the effect on succinic acid production. Carbohydrates can be consumed by *E. coli* for the fermentation, so glucose was added to all the samples to see the nutritional and inhibitory effects of the AP-bio-oil on the succinic acid production. Dual-phase fermentation was adopted, which includes an aerobic phase for cell production and an anaerobic phase for succinic acid production. Figure 3 (a) presents the glucose consumption in the aerobic phase which reflects the bacterial growth. Figure 3 (b) shows the glucose concentration in the anaerobic phase which reflects the succinic acid production. Figure 3 (a) shows that the glucose was depleted only in the media with Fermentation components which means that the bacterium require these for growth. For the media with AP-bio-oil concentrations below 25 v/v%, the glucose concentration decreased similarly which means that the bacterial growth was unaffected. All these results were consistent with the results showed in Figure 2 (b). The interesting thing is that there is not so much glucose consumed in the media with 50–100 v/v% of AP-bio-oil and Fermentation components (FC). However, Figure 2 (b) indicated that the strain in these two media grew well since the final OD value was 1.58 and 1.9 for the media with 50 and 100 v/v% of AP-bio-oil, respectively and FC. One speculation for this phenomenon is that the stain grows by using the nutrition from the AP-bio-oil.

Figure 3 (c) shows succinic acid concentration in the anaerobic phase of the fermentation process. A similar amount of succinic acid (51–52 g/L) was produced in the media with less than 12.5 v/v% AP-bio-oil and FC. The final succinic acid concentrations for the media with v/v% of AP-bio-oil on 25, 50 and 100 were 47 g/L, 23 g/L, and 6.4 g/L, respectively. From this it seems like as the AP-bio-oil percentage increased to more than 12.5 v/v%, the succinic acid production decreased. This should attribute to the small amount of cells since there were fewer cells produced in the aerobic phase. Another reason is that the inhibitory compounds existing in the AP-bio-oil impeded the succinic acid fermentation. The key results of this fermentation are shown in Table 2.

**Table 2:** Succinic acid production from corn stover pretreated by enzymatic hydrolysis and thermochemical method

**AP-bio-oil conc (%)**	**Succinic acid (g/L)**	**Glucose consumed (g/L)**	**Xylose consumed (g/L)**	**Succinic acid yield (g/g sugar)**
0	10.7	36.1	5.0	0.26
2.5	10.9	38.3	5.3	0.25
5	10.9	38.3	5.3	0.25
12.5	10.9	34.4	4.6	0.28
25	9.5	36.6	4.7	0.23
50	7.6	37.3	4.9	0.18
STDEV	0.6	3.7	0.7	0.02

### AP-Bio-oil changes during the fermentation process

**Figure 4:**
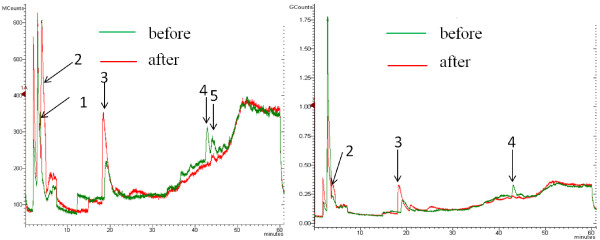
**GC-MS analysis of AP-bio-oil components before and after fermentation. a**: fermentation medium with 50% AP-bio-oil; **b**: fermentation medium with 100% AP-bio-oil. Peaks for possible components: **1**:3-methyl-butanal; **2**: ethanol; **3**: acetic acid **4**: 2, 3-dihydro-3, 5-dihydroxy-6-methyl-4H-Pyran-4-one; **5**: dimethoxy phenol.

The AP-bio-oil components before and after the fermentation were analyzed by GC-MS as shown in Figure 4. The peak of acetic acid got higher during the fermentation because this is a byproduct in the fermentation. The original acetic acid in the bio-oil was used during the fermentation process. Besides acids, some other low-molecular-weight compounds such as butanal compounds (Figure 4 (a)) can be nutritive. The peaks of 2, 3-dihydro-3, 5-dihydroxy-6-methyl-4H-Pyran-4-one in both Figure 4 (a) and (b) were diminished which indicated that this compound was used. In the medium with 50 v/v% bio-oil, dimethoxyphenol compounds was used or decomposed as indicated from the disappeared peak. In Figure 4 (a), most peaks disappeared and some peaks such as the peak of ethanol appeared because ethanol is a byproduct in the fermentation. Less peak changes were observed in the medium of 100 v/v% AP-bio-oil. It can be concluded that component changes of 50 v/v% AP-bio-oil varied sharply compared to that of 100 v/v% AP-bio-oil. This should be attributed to the different fermentation results. Succinic acid concentrations were 23.3 and 6.4 g/L in the media with 50 and 100 v/v% of bio-oil, respectively.

### Fermentative production of succinic acid with AP-bio-oil and enzymatic hydrolyzed biomass

**Figure 5:**
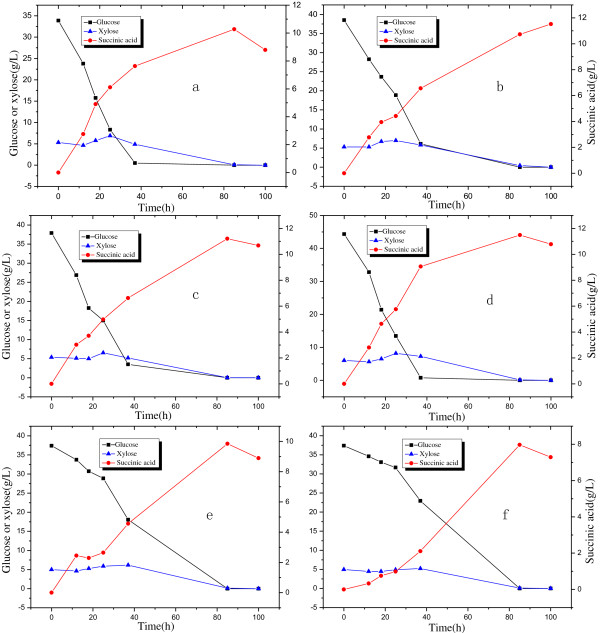
**Production of succinic acid with biomass pretreated by both biotechnological and thermal process.** AP-bio-oil concentration: (**a**) 0%; (**b**) 2.5%; (**c**) 5%; (**d**) 12.5%; (**e**) 25%; (**f**) 50%.

Succinic acid was produced from AP-bio-oil mixed with glucose which was derived from corn stover treated with enzymatic cellulose hydrolysis. This part of the experiment was conducted according to condition 3 in Table 3 at AP-bio-oil concentrations between 0 and 50 v/v%. Figure 5 presents succinic acid, glucose and xylose concentration during this fermentation process. Table 2 shows succinic acid concentration and yield, and the amounts of consumed glucose and xylose. From these results, the highest succinic acid concentration increased versus the bio-oil concentration in the range 0 to 12.5 v/v% from 10.3 to 11.5 g/L. By further increase in bio-oil concentration to 50 v/v%, succinic acid concentration decreased to 8.0 g/L. Inhibition was thereby obvious when the bio-oil concentration was higher than 25 v/v%, which is reasonable since AP-bio-oil contain phenol, aldehydes, ketones and pyran compounds. Glucose and xylose derived from the biomass were used up at the end of the fermentation. From Table 2, the highest Succinic acid concentration of 10.9 g/L was obtained with 12.5 v/v% AP-bio-oil. Thus, AP-bio-oil can promote bio-production of succinic acid, and contribute to the chemical production platform.

**Table 3:** Experiments design of succinic acid fermentation with AP-bio-oil and steam exploded corn stover showing the maximum succinic acid concentrations (g/L)

**AP-bio-oil conc %**	**Combination 1 + FC + glucose**	**Combination 2 -FC + glucose**	**Combination 3 -FC + CS**
0	52.0	0	10.3
2.5	-	0	10.7
5	-	0	11.2
12.5	50.9	0	11.5
25	47.2	0	9.8
50	23.3	0	8.0
100	6.4	0	-

*FC* = Fermentation components, *CS* = Steam exploded corn stover.

“-”means experiments in this combination wasn’t conducted.

## Conclusion

The AP-bio-oil can provide carbon source and little nitrogen source to support the growth of *E. coli* MG-PYC. Bacteria can grow in AP-bio-oil with some mineral and nitrogen salts added while there is no growth when nothing was added. Furthermore, in the traditional fermentation media, the bacterium grew much better. Fermentation results revealed that it is possible to use AP-bio-oil as source of chemical feedstock. In the modified M9 media, the final succinic acid concentration increased versus AP-bio-oil concentration. With Fermentation components added, the highest succinic acid was achieved when AP-bio-oil concentration was lower than 12.5 v/v%. It is not advisable to use high AP-bio-oil concentrations because of the existence of inhibitory compounds in the AP-bio-oil. It is of significant importance that succinic acid was produced from biomass treated by both a thermal process and a biotechnological process. Biomass derived AP-bio-oil and glucose was used for succinic acid production and the best fermentation result was obtained when the AP-bio-oil percentage was 12.5 v/v%. GC-MS analysis indicates that acids and some other low-molecular-weight compounds were utilized. Some pyran and phenol compounds can also be used or decomposed during the fermentation. This is the first exploration to see the possibility of AP-bio-oil usage for production of succinic acid. Some positive results were achieved, providing another possible way of AP-bio-oil as a chemical production source.

## Materials and methods

### Strain and growth conditions

The bacterial strain *E.coli* MG-PYC was constructed by transformation of the plasmid pTrchisA-*pyc* into *E.coli* MG1655. In *E.coli* MG1655, the *ldhA* gene involved in the lactic acid synthesis pathway is deleted for increased succinic acid production. During the strain construction, the cultures were grown aerobically at 37°C in Luria + Bertani (LB) medium (10 g tryptone, 5 g yeast extract, and 5 g NaCl per Liter). Solid media for plates contained in addition15 g/L Bacto agar. Antibiotics were included as necessary at the following concentrations: 34 mg/L Ampicillin ((2S,5R,6R)-6-([(2R)-2-amino-2-phenylacetyl]amino)-3,3-dimethyl-7-oxo-4-thia-1-azabicyclo[3.2.0]heptane-2-carboxylic acid) and 30 mg/L Kanamycin (2-(aminomethyl)- 6-[4,6-diamino-3-[4-amino-3,5-dihydroxy-6-(hydroxymethyl) tetrahydropyran-2-yl]oxy-2-hydroxy- cyclohexoxy]- tetrahydropyran- 3,4,5-triol).

The Fermentation components contained per liter were initially: 0 g/20 g glucose, 20 g tryptone, 10 g yeast extract, 0.15 g MgSO_4_, 0.2 g CaCl_2_, 0.02 g MnCl_2_, 0.45 g Na_2_HPO_4_ · 12H_2_O, 6 g NaH_2_PO_4_ · 2H_2_O and 3 g (NH_4_)_2_SO_4_ · 7H_2_O. pH was adjusted to 7 with NaOH. IPTG (Isopropyl-β-D-thio-galactoside) was added at 23.8 mg/L to the medium to induce gene expression of Phosphoenolpyruvate (PEP) carboxylase (ppc) for plasmid pTrchisA-*pyc*. The chemicals used were of analytical grade and purchased from either OXOID (England) or Sinopharm Chemical Reagent Beijing Co., Ltd (China) unless otherwise described.

### Production of AP-bio-oil

The bio-oil was produced from rice husk by fast pyrolysis at 550°C [YINENG Bio-energy Company, Shandong province, China]. Bio-oil was separated into an aqueous phase and an organic phase by adding 20 g of water per g of bio-oil. After adding water, the liquids were stirred for 20 minutes and then centrifuged at 8000 rpm for 25 min. Solids were separated and AP-bio-oil was obtained for the experiments included in this study. The concentration of the carbon, nitrogen and hydrogen in the AP-bio-oil in this study is 1.26%, 0.5% 8.35%, respectively.

### Strain growth in M9 medium with AP-bio-oil

The modified M9 medium with different concentrations of AP-bio-oil was used to test whether this can provide carbon source or nitrogen source. The M9 medium contained carbon source (4 g glucose), nitrogen source (1 g NH_4_Cl) and mineral salts (3 g KH_2_PO_4,_ 6 g Na_2_HPO_4_ · 12 H_2_O, 0.12 g MgSO_4_ and 0.5 g NaCl) per liter (Table 1). Medium 1, 3 and 4 were designed to see whether AP-bio-oil can act as carbon source. Medium 2 was used to detect whether acetic acid can be used as carbon source since AP-bio-oil contains acetic acid. Medium 5–8 were used to test if the AP-bio-oil can act as nitrogen source.

### Succinic acid fermentation with AP-bio-oil and fermentation components

The experimental design for the fermentation is shown in Table 3. The succinic acid fermentation was performed in 250 mL flask containing 100 mL fermentation medium and 5 v/v% inoculum of the transformed *E.coli* MG1655 obtained from the LB plate colonies. The fermentation medium composition is described in Part 4.1. The strain was cultivated aerobically at 220 rpm and 37°C for 12 h. Then MgCO_3_ was added to the flasks as a CO_2_ source and to control pH at 6.7. The head space was filled with CO_2_ to start the anaerobic inoculation at 37°C for 195 h. Sterilized solution of 50 w/w% glucose was added intermittently after the beginning of the anaerobic phase.

### Fermentation of glucose from corn stover mixed with AP-bio-oil

Steam exploded corn stover was produced in a reactor of 4 L volume [ZhengDao Company, HeNan province, china]. The temperature was gradually increasing from 190 to 220°C during the residence time of 4 ± 1 min. Enzymatic hydrolysis was performed with 100 g/L of dry matter (DM) with cellulase enzymes at 30 FPU (Filter paper units)/g DM at pH 5.0 for 60 h followed by autoclaving at 115°C for 30 min [XIASHENG Company, NingXia province, China]. A high density culture stock (OD = 20) was obtained by aerobic fermentation in LB medium at 37°C for 12 h followed by centrifugation at 5000 rpm for 10 min and resuspension of the pellet in water. The culture stock was added to obtain an OD of 4 and the anaerobic fermentation was performed for 100 h.

### Analytical methods

The bacterial growth conditions were estimated from the optical density (OD) of the medium with a spectrophotometer (723 N, Shanghai Precision & Scientific Instrument Co. Ltd, China) at a wavelength of 600 nm. The concentrations of glucose and organic acids were analyzed by high performance liquid chromatography (HPLC), Agilent1200 [Agilent, Co. Ltd USA] equipped with UV absorbance and refractive index detectors and a Bio-Rad Aminex HPX-87H column (300 × 7.8 mm). The mobile phase was 5 mmol/L of H_2_SO_4_, the flow rate was 0.6 mL/min and the column temperature was 50°C. Samples of culture broth (1 mL) were taken and centrifuged at 10000 rpm for 10 min. The supernatant was diluted 10 times, and 10 μL of the diluted sample was injected into the HPLC and 0.01 μL into the GC-MS (gas chromatography - mass spectrometry). The GC-MS equipment Varian CP-3800/300-MS was used with a capillary column of FFAP (30 m - 0.25 mm - 0.25 μm). The oven temperature started at 40°C (3 min) and then increased to 100°C (3 min) by 4°C/min and finally increased to 220°C (9 min) by 4°C/min.

## Abbreviations

AP-bio-oil: Aqueous phase bio-oil; GC-MS: Gas chromatography - mass spectrometry; HPLC: High performance liquid chromatography; OD: Optical density; rpm: Rounds per minute

## Competing interests

The authors declare that they have no competing interests.

## Authors’ contributions

Caixia Wang and Anders Thygesen carried out the bacterial construction, bacterial growth, biomass hydrolysis, fermentation, data analysis and drafted the manuscript. Yilan Liu deleted the *ldhA* genes from wide MG1655. Qiang Li and Maohua Yang participated in its design and coordination and helped to draft the manuscript. Dan Dang and Ze Wang carried out GCMS analysis. Yinhua Wan,Weigang Lin and Jianmin Xing conceived of the study, and participated in its design and coordination and helped to draft the manuscript. All authors read and approved the final manuscript.
